# Risks of Antibiotic Exposures Early in Life on the Developing Microbiome

**DOI:** 10.1371/journal.ppat.1004903

**Published:** 2015-07-02

**Authors:** Anjelique Schulfer, Martin J. Blaser

**Affiliations:** 1 Departments of Medicine and Microbiology, New York University Langone Medical Center, New York, New York, United States of America; 2 VA Medical Center, New York, New York, United States of America; 3 Department of Biology, New York University, New York, New York, United States of America; University of North Carolina at Chapel Hill School of Medicine, UNITED STATES

During birth, a relatively sterile unborn child becomes a newborn coated with microbes on every surface. This collection of bacteria, archaea, viruses, and fungi found in and on the human body is called the microbiota. The collective genomes of the microbiota are considered to be the metagenome, and the totality of the microbiota, metagenome, and their interactions is the microbiome. The microbiota has many critical functions including protection from pathogens, development and maintenance of the immune system, and helping the host access nutrients in food. The gut microbiota has been of particular interest, as perturbations of this community have been linked to disease states including autoimmune disease and neurological disorders. Antibiotics have consistently been shown to change the gut microbiome in humans and animals. We briefly review the importance of the early-life gut microbiome and the risks of disturbing it with antibiotics.

Each method used to measure changes in the microbiota and microbiome has advantages and disadvantages. Culturing provides reliable results but is labor intensive and limited in the number of identifiable taxa. 16S rRNA gene (16S) sequencing can efficiently identify taxa; however, the results depend on the accuracy of 16S sequence databases. Whole genome sequencing (WGS) is prohibitively expensive for most experiments; however, it can describe both taxa identity and genes available in the community. Predictive metagenomics based on 16S sequencing can estimate genes available in the community, but they are not directly measured. Metabolomics examines which molecules are changing in the bacterial community that could impact the host. These methods are all currently in use and those changes most important for host physiology and susceptibility to disease are not known.

## How Stable Is the Early-Life Gut Microbiota?

Delivery mode initially determines the neonatal gut microbiota, with babies born vaginally having a microbial profile resembling their mother’s vagina and cesarean section babies getting their first microbes from the skin of people who contact them [1]. This initial microbiota has very low diversity and high instability compared to microbiota profiles later in life, making it susceptible to disturbances such as illness, antibiotic treatment, and dietary changes [2,3]. Ecological analysis of the gut microbiota of a single infant over the first 2.5 years of his life using both 16S sequencing and WGS showed that microbial community succession over this time was nonrandom. This indicates that the starting point of the gut microbiota will influence the communities found later in life [2]. The early-life time frame is especially important because we know that the immune system is still developing after birth and is in part shaped by the gut microbiota [4]. Unsurprisingly, it has been shown that the effects of delivery mode on the microbiota can be long-lasting and have an impact on the health of the child. For example, children born by cesarean section have been shown to be at higher risk for some immune related disorders [1].

The infant gut microbiota increases in diversity and richness while becoming more stable over time, especially once solid foods are introduced into the diet, until the community resembles an adult-like state at around three years old [1,3]. The initial microbiota and community succession are unique to each infant, influenced by host genetics, and susceptible to disturbance [1,5]. This makes the infant gut microbiota vulnerable to changes that may have lifelong health implications.

## What Effects Do Antibiotics Have on the Microbiota and Does the Microbiota Recover?

The exact impact of antibiotics on the human gut microbiota has been difficult to pinpoint because each individual’s response is unique [6,7]. However, several key studies clearly indicate that antibiotic treatment changes the gut microbiota communities identified by 16S sequencing, at least transiently. After seven days of clindamycin treatment, patients had significantly reduced diversity of the *Bacteroides* community compared with the pretreatment microbiota that remained distinctive two years after antibiotic cessation [8]. Similarly, in addition to decreasing bacterial diversity, a five day course of ciprofloxacin changed relative abundance of 30% of the gut microbiota community members. To a great extent, these communities recovered to pretreatment levels by four weeks postantibiotic; however, some changes lasted at least six months, including the loss of particular Clostridiales [6]. In a follow-up study, a second exposure to ciprofloxacin was associated with highly variable responses between individuals, often with changes in the gut microbiota similar to those from the first exposure with less complete recovery after antibiotic cessation [7].

Currently, it is not possible to assess how important losses of specific bacteria are to an individual. That human antibiotic trials often have no gastrointestinal symptoms accompanying the gut microbiota changes suggests functional redundancy in the community [6]. However, in mice, antibiotics can significantly impact the metabolic pathways functioning within the gut microbiota; a single streptomycin dose given to mice decreased the number of fecal bacteria by 90% by 12 hours with recovery to pretreatment levels taking 6 days. At 24 hours after streptomycin treatment, 87% of detectable metabolites in the feces had been qualitatively or quantitatively altered, including a decrease in 17 metabolites involved in the synthesis of primary bile acids. This suggests that this single antibiotic dose had substantially changed the biochemistry of the gut [9].

**Fig 1:**
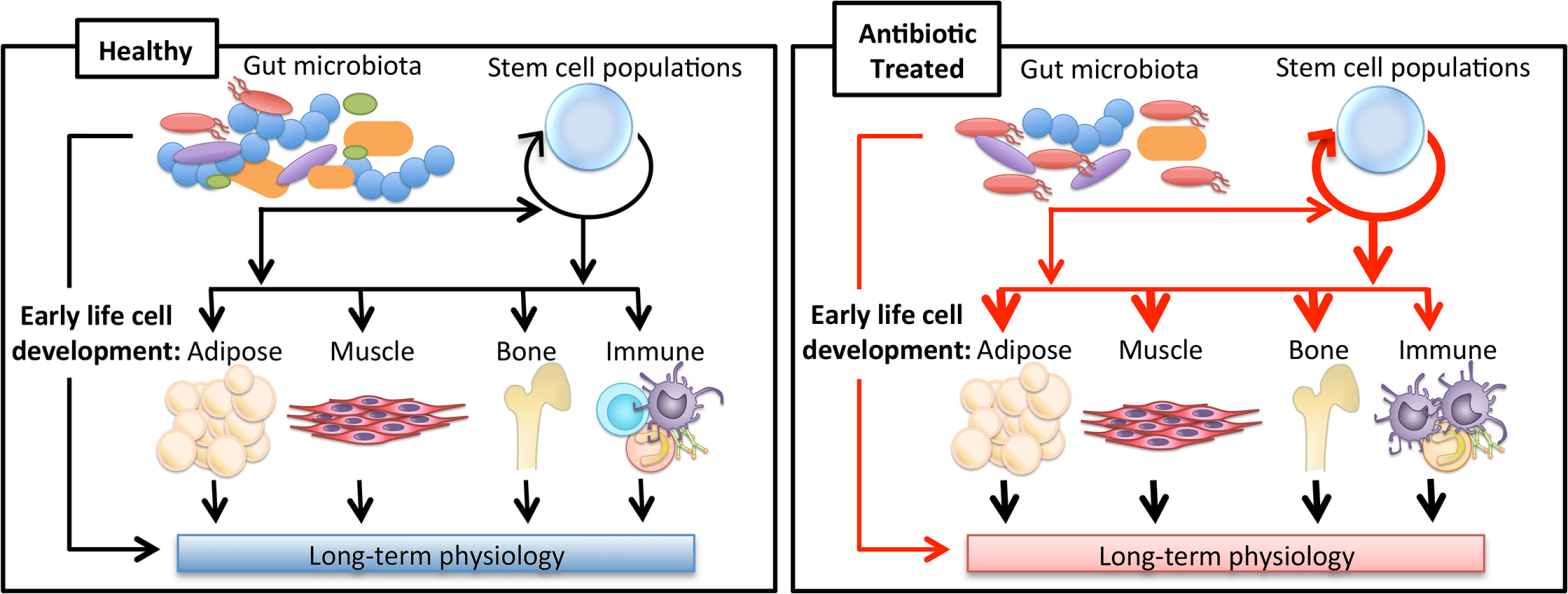
Impact of antibiotics on long-term physiology through microbiota changes. The gut microbiota has been shown to influence the development of the host’s immune system in addition to being implicated in adipose, muscle, and bone tissue growth. New evidence indicating the gut microbiota may impact stem-like cell populations suggests a new way in which the gut microbiota may be regulating tissue development. Antibiotics alter the gut microbiota, which may change the course of these developmental pathways, leading to variations in long-term physiology.

Most studies, such as those described above, have not specifically investigated early-life antibiotic exposure. They give an idea of the power of antibiotics on the microbiota, and from other studies we can see the importance of the early life window. 16S sequencing has shown that mice exposed to low-dose penicillin before weaning had an altered gut microbiota compared to controls, including a decrease in *Lactobacillus* and *Allobaculum*, but the community composition recovered to resemble the control communities after the antibiotics were removed. Importantly, despite microbiota recovery, the mice that were antibiotic-exposed in early life had increased fat, lean, and total mass even 26 weeks after the antibiotic treatment was stopped [10]. The increase in mass was only seen when antibiotics started before weaning. Such observations are consistent with a role of the microbiota in a developmental window for host metabolism. A potential interaction schema by which antibiotic use, perturbing the microbiota, alters crosstalk with developmental networks determining cell fate, leading to long-term consequences, is illustrated in Fig 1.

The importance of this early-life window in determining host health has also been established with regard to the microbiota and immunity. Germ-free (GF) mice, which have never been exposed to microbes, have impaired immune function including increased serum IgE levels, which is a hallmark of autoimmune disorders. Normal IgE levels can be restored by colonizing GF mice with a healthy mouse’s gut microbiota, but only if the microbes are given early in life [11].

The gut microbiome can be a reservoir of antibiotic resistance genes, which is another important consideration of the impact of antibiotics on the microbiota. Patients with gastric or duodenal ulcers who received a standard treatment of metronidazole, clarithromycin, and omeprazole for seven days had levels of *erm*(B), a macrolide resistance gene, 1,000 times higher than control patients; in the absence of any further known selection, elevated levels of this antibiotic resistance gene persisted for at least four years [12]. It is thus becoming clear that antibiotics can select for altered community composition, function, and antibiotic resistance of the gut microbiome. The extent of recovery to pretreatment levels is dependent on a mix of factors including the nature of the antibiotic, the duration of treatment, as well as intrapersonal characteristics (including host genetics), and probably microbiota composition.

## What Are the Health Implications of an Altered Gut Microbiota?

Since the gut microbiota is important in host immune development, nutrient absorption, and protection from pathogens, changes in the community composition could have deleterious effects on the host. The spectrum of diseases for which an altered gut microbiota has been implicated is quite broad. Several excellent reviews have focused on the relationship between the microbiota and host immunity. One critical aspect of this field is that T cell populations in the gut can be influenced by the microbiota and its metabolites. One of the most studied groups of bacterial metabolites, short-chain fatty acids, have been shown to exert epigenetic regulation of transcription factor genes to influence regulatory T cells in the gut [13]. Changes in T cell populations are one mechanism by which alterations in the gut microbiota may be contributing to autoimmune diseases including inflammatory bowel disease (IBD), asthma, allergies, arthritis, and multiple sclerosis (MS) [4,14].

While much of the evidence to date suggesting a link between the gut microbiota and autoimmune disease comes from differences in pathology between GF and conventionalized mice, there are numerous human association studies to suggest that such findings are applicable to humans [4,14]. In children with new-onset Crohn’s Disease, antibiotics amplified the changes in the microbiota and caused a mild increase in disease severity. 16S sequencing revealed that antibiotics in these children triggered a major reduction in taxa including Bacteroidales and Erysipelotrichaceae, which are typically associated with noninflammatory conditions [15]. At least nine studies based on questionnaires or database searches have shown an increased likelihood of IBD diagnosis in people who have taken antibiotics [16]. Although these studies have mostly focused on children, there is limited evidence that adults who receive antibiotics have an increased chance of developing IBD within 2–5 years [16]. Similar database searches have shown that antibiotic use is associated with an increased risk of diabetes, childhood asthma, and a 30% higher risk of MS development [17,18,19]. Though correlative studies such as these cannot prove causation, they provide collateral evidence for the hypothesis that an altered microbiota can contribute to disease, which is well supported by mouse studies.

Antibiotic use early in life has been shown to change the gut microbiota in mice and lead to increased weight gain in both mice and humans [10,20]. Antibiotics also can influence the host directly. A transfer experiment using GF mice showed the increased weight gain in mice was due to microbiota changes, not antibiotic exposure [10]. Antibiotic use also increases susceptibility to infection [21]. In the case of *Clostridium difficile*, this can prove fatal. Currently the treatment with the highest cure rate for *C*. *difficile* infection, >90% in many trials, is fecal microbiota transplant (FMT) [22]. Recent discoveries showing the influence of the gut microbiota on stem cells and brain function suggest that the list of disorders associated with an altered early-life gut microbiome will continue to expand [23,24]. Moving beyond database correlations and small clinical trials to determine causal roles in humans is a major challenge facing researchers in the field.

## Can the Impact of Early-Life Antibiotic Exposures Be Mitigated?

There is no question that antibiotics have revolutionized medical care and continue to be essential to our society. While we should strive to limit unnecessary antibiotic exposure and improve diagnostics to allow for more effective use of narrow-spectrum antibiotics with fewer off-target effects, there always will be situations in which antibiotics must be used, especially early in life. For such cases, we need to understand which microbes and metabolic pathways are important for a healthy microbiome if, after disturbance by antibiotics, we hope to manipulate gut communities with prebiotics and/or probiotics. This is highly challenging since, as discussed above, the human gut microbiota is extremely variable between individuals and over time, especially in the first few years of life.

More robust clinical studies are needed to assess the short- and long-term impacts of antibiotic exposures on the gut microbiome. These studies need to be adequately powered and utilize WGS to enable drawing relevant conclusions. Such focus, which also may be age-specific, can lead to targets for reconstitution. It may soon prove prudent for fecal samples to be collected from children before taking antibiotics, in case they need to recover beneficial microbes with an FMT [4]. Interest in the off-target (collateral) impact of early-life antibiotics on the microbiome has been relatively recent. Although we currently lack research-based solutions to mitigate the impact of antibiotic treatment early in life, we are hopeful that investigation over the next several years will provide deeper understanding into this newly recognized problem, thus leading to preventives and treatments.
